# Childhood leukaemia and population movements in France, 1990–2003

**DOI:** 10.1038/sj.bjc.6604141

**Published:** 2007-12-18

**Authors:** S Bellec, B Baccaïni, A Goubin, J Rudant, M Ripert, D Hémon, J Clavel

**Affiliations:** 1 INSERM, U754, Hôpital Paul Brousse, F-94807 Villejuif, France; 2 Université Paris XI, F-94276 Kremlin-Bicêtre, France; 3 French Registry of Childhood Haematopoietic malignancies, F-94807 Villejuif, France; 4 INSEE Provence-Alpes-Côte d’Azur, F-13387 Marseille, France

**Keywords:** childhood leukaemia, population movements, population density, infectious aetiology hypothesis

## Abstract

In a national study, we investigated the incidence of childhood leukaemia (CL) over a 14-year period in France in relation to several measures based on the proportion of individuals who changed address between the last two national censuses. A positive association was found with the proportion of migrants who came from a distant place. The further the migrants came, the higher was the incidence of leukaemia, particularly among children aged 0–4 years in ‘isolated’ *communes* at the time of diagnosis (RR=1.4, 95% CI: 1.1,1.8 in the highest category of migration distance). Although the role of the population density was less obvious, a more marked association was found above a certain threshold. No association with the proportion of commuters was observed.

About 20 years ago, a cluster of cases of childhood leukaemia (CL) in the village of Seascale (England) revived interest in an infective basis by leading to the population mixing hypothesis (Kinlen, 1988). This proposed that large population influxes into rural or isolated areas are conducive to an epidemic of the underlying infection(s) of which CL is postulated to be a rare consequence. Childhood leukaemia mortality or incidence has been examined in rural areas after receiving such influxes (Kinlen, 1988, 2006; Kinlen *et al*, 1990, 1993, 2002; Kinlen and Hudson, 1991; Langford, 1991; Kinlen and John, 1994; Kinlen and Petridou, 1995; Koushik *et al*, 2001; Boutou *et al*, 2002). These studies, mainly focusing on historically documented and specific rural population increases (with consequent increases in population density), showed significant increased relative risks, in the range of 1.5–4.7, in the places with the highest population increases, compared to the reference group.

Elsewhere, population mixing has mainly been defined not as population increases but as increases in the proportion of residents who changed address over a specified period, or in the year before a census, and not specifically in rural areas (Stiller and Boyle, 1996; Dickinson *et al*, 2002; Parslow *et al*, 2002; Law *et al*, 2003; Labar *et al*, 2004; Nyari *et al*, 2006; Rudant *et al*, 2006). Overall, the studies seem to favour a positive association between the incidence of, or mortality associated with, CL and the highest proportions of migrants, although a few have reported a negative association (Parslow *et al*, 2002; Law *et al*, 2003). A few of these also found that the further the migrants came, the higher was the incidence of CL (Stiller and Boyle, 1996; Dickinson *et al*, 2002; Rudant *et al*, 2006). In the past, a positive association was found with childhood acute lymphoblastic leukaemia (ALL), particularly in isolated areas with a population density greater than a given threshold at the time of birth.

In the present study, we investigated CL incidence on a national scale in relation to the proportion of individuals who changed address over a 14-year period, focusing on the isolation status of residence at diagnosis.

## MATERIALS AND METHODS

In 1999, mainland France consisted of 36 565 *communes*, the smallest administrative unit, 95 *départements* and 22 *régions*. Owing to commune merging or splitting before 1999, information was sometimes available only for merged *communes*. The whole of the country was finally divided into 36 347 *communes* or combinations thereof, which, for simplicity, are still referred to as *communes*. This ecological study was conducted on the national scale in France. All cases of CL registered in the French National Registry of childhood haematopoietic malignancies (Clavel *et al*, 2004) and diagnosed between 1990 and 2003 were included. Each case was associated with its *commune* of residence at the time of diagnosis.

To define the status of each *commune* in 1990, two classifications by the French National Institute for Statistics and Economic Studies (INSEE) were considered, the Urban Zoning Classification and the Urban Unit classification (see Rudant *et al*, 2006). From the former, based on the influence and dependence in terms of employment, ‘attracting’ *communes* were those that attracted a substantial number of commuters from other *communes* for jobs. On the basis of this classification, ‘dependent’ *communes* were then defined as those with at least 40% of their economically active population working outside, in ‘attracting’ *communes*. Overall, ‘attracting’ and dependent *communes* accounted for about 9 and 29% of *communes*, respectively.

The Urban Unit classification classified the French *communes* by the population size of the Urban Unit, defined as a group of *communes* in which the distance between dwellings was not more than 200 m.

On the basis of those criteria, a *commune* was then considered *a priori* ‘isolated’ if it belonged to an Urban Unit with a population of less than 5000 or to a rural *commune*, and was neither an ‘attracting’ nor ‘dependent’ *commune* in 1990. A few *communes* called ‘urban’ by INSEE were therefore included in the ‘isolated’ *commune* group, but they are small, not ‘attracting’, not ‘dependent’ and situated in remote areas. The ‘isolated’ *communes* accounted for 61% of *communes* and 18.5% of French population in 1990.

The population movements between the last two national population censuses were used as proxy measures of population mixing. The number of individuals who moved from a *commune* to another between 1990 and 1999 was the initial focus. To take account of the distance covered by the migrants, the numbers of people in a *commune* outside the *département*, outside the *région* or in a distant *commune* in 1990 were considered. A *commune* was considered distant from another, defined *a priori*, if at a greater distance than the median distance covered by the migrants between 1990 and 1999 (100 km overall, 60 km for ‘isolated’ and 120 km for ‘non-isolated’ *communes*). All these measures were considered as proportions of the 1999 population.

We also considered the weighted average migration distance *d*_*i*_ defined by 
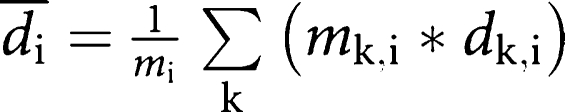
, in which *m*_*ki*_ is the number of migrants who moved from a *commune k* to given *commune i*, *m*_*i*_ the total number of immigrants in *commune i* and *d*_*ki*_ the distance between the *communes i* and *k*. This measure was introduced in regression models with adjustment for the overall proportion of migrants. Lastly, the proportion of regular commuters was evaluated, from the 1999 census data, as the sum of economically active people in a given *commune* but working outside it and those living outside it but working in it, divided by the 1999 population of the *commune*.

### Statistical analyses

The INSEE provided population estimates by *commune* for the two census years 1990 and 1999, and population estimates by *département* from 1990 to 2003 (a French *département* consists of about 385 *communes* on average). The populations between the two censuses and after the year 1999, on the *commune* scale, were then estimated as follows: 
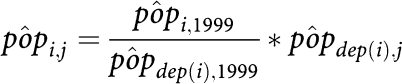
 in which *i* and *j* are the index numbers for a given *commune* and a given year, respectively, *pôp*_*i*,*j*_ is the population estimate for *commune i* in year *j* and *pôp*_dep(*i*),*j*_ is the population estimate for the relevant *département* for the year *j* (source INSEE).

The numbers of CL cases expected in each French *commune* over the study period 1990–2003 were based on the population estimates and the national 5-year age-specific incidence rates provided by the RNHE.

The associations between the various measures of population movements and CL incidence were investigated using Poisson regression models. Each variable was incorporated in the statistical models as a three-category variable, the breakpoints being chosen *a priori* on the basis of the quintile distribution of the expected numbers, to isolate the highest quintile. The first two groups were then defined so that they each included 40% of the total number of expected cases. The Poisson regression models estimated the SIRR, the ratio of the SIR (standardised incidence ratio) for a category and that of the baseline category. Cut points were defined separately for all, ‘isolated’ and ‘non-isolated’ *communes*. However, when variables varied only slightly with isolation status, in terms of distribution of the expected cases, common cut points for the various groups were adopted.

Additional analyses were stratified by age (0–4, 5–9 and 10–14 years), isolation status of the *communes* (‘isolated’, ‘non-isolated’) and population density. The latter was transformed into a three-category variable, each group containing similar expected numbers at ages 0–14 years, whereas the ‘isolated’ group was only split at the median because of the lower expected numbers. The breakpoints were then 180 and 1729 inhabitants per square kilometre (inh km^−2^) in the overall analyses, 394 and 2607 inh km^−2^ in the ‘non-isolated’ group and 45 inh km^−2^ in the ‘isolated’ group. The analyses of weighted average distance of migration were systematically adjusted for the total proportion of migrants. Lastly, some analyses were performed for common B-cell lymphoblastic leukaemia (common B-cell ALL).

All analyses were performed with the SAS® and R software.

## RESULTS

Mainland France was divided into 36 347 *communes*, 22 252 of which were ‘isolated’ (60%) and inhabited by only 18.5% of the total French population, which reached 56.6 million in 1990 (58.5 million in 1999), with about 20% aged up to 14 years. C*ommune* populations range from 1 to more than 2 000 000 inh, with median and mean of about 350 and 1500 inh, respectively. Similarly, population density ranged from 1 to over 22 000 inh km^−2^ (median 33). As expected, the ‘isolated’ *communes* were characterised by a smaller population and a lower density.

Overall, 19 657 175 people changed address between the two censuses, 18 336 140 coming from a *commune* in mainland France of known isolation status. About 18% of the migrants moved to an ‘isolated’ *commune*, the median distance covered being about 100 km overall, 60 and 120 km for ‘isolated’ and ‘non-isolated’ *communes* of destination, respectively (Table 1). Overall, half the *communes* were subject to a total population influx of between 26 and 38% of their 1999 population (Table 1). ‘Non-isolated’ *communes* had a slightly greater proportion of migrants than ‘isolated’ *communes*, the median proportions from another *département* or *région* being 11.4 and 7.8%, respectively, with slightly higher proportions in ‘isolated’ than in ‘non-isolated’ *communes*. The proportions of migrants in a distant *commune* in 1990 were mainly below 10% and were slightly higher in ‘isolated’ *communes*. These showed a higher weighted average migration distance than the ‘non-isolated’ *communes* (Table 1). Lastly, half the *communes* had between 30.5 and 45% regular commuters, and the proportions were markedly greater in the ‘non-isolated’ group.

Of the 6288 children aged 0–14 years with CL in the National Registry of Childhood Haematopoietic malignancies between 1990 and 2003, *commune* of residence at diagnosis was missing for 14 (0.2%) leaving 6274 in the present analyses, half aged less than 5 years at diagnosis. A total of 4090 cases of common B-cell ALL were registered over 1990–2003, 65% of the total. Overall, 5220 cases of CL (83% of the total) were diagnosed in a ‘non-isolated’ *commune*.

Overall, no association with the proportions of all migrants, of migrants from another *département* or of commuters was found (Table 2). However, an increased incidence was observed with the proportion of migrants who came from another *région*, particularly in the ‘isolated’ *commune* group, a proportion greater than 13% being associated with a significant 18% increase in CL incidence. Similarly, there was a slight increase in incidence in the group with the highest proportions of migrants from distant *communes* (SIRR=1.08 [1.02; 1.16]). This was seen in both isolation groups, greater in ‘isolated’ *communes* although of borderline significance (SIRR=1.14 [0.97; 1.33]). Adjusting for the total proportion of incomers, the weighted average migration distance was weakly associated with CL incidence, more marked for the ‘isolated’ *communes* (SIRR=1.17 [1.02; 1.34] for a distance in the range 82–185 km and 1.24 [1.02; 1.5] for a distance greater than 185 km).

Overall, most of the above results were found, mostly reinforced, in children aged 0–4 years (Table 3). The highest proportion of migrants from another *région* was associated with a 20% increase in CL incidence. In the ‘isolated’ *commune* group, more than 12% of migrants from a distant commune showed a 25% increase in CL (SIRR=1.25 [1.01; 1.56]). Similarly, allowing for the total proportion of migrants, the ‘isolated’ *communes* with a weighted average migration distance of more than 185 km were at a significant increased risk (SIRR=1.41 [1.09; 1.83]). Some of the associations were still observed in the 5- to 9-year age group, but none above that age group (results not shown).

When stratifying by the population density, there was no association with the total proportion of migrants (results not shown) but a nonsignificant increase was found in the ‘isolated’ *communes* with a population density below 45 inh km^−2^ (SIRR=1.28 [0.91; 1.79] for proportions of migrants above 38%).

Considering all *communes*, whatever the proxy measure, based on the migration distance – from another *région*, or a distant *commune* or weighted average distance – the positive association with CL incidence at ages 0–4 years seemed restricted to low-density *communes* (⩽180 inh km^−2^; Table 4). Those *communes* showed a more than 20% CL increase in the highest category (SIRR=1.27 [1.07; 1.51] for proportions of migrants from another *région*, SIRR=1.22 [1.03; 1.45] from a distant *commune* and SIRR=1.28 [1.04; 1.58] for the weighted average migration distance).

In the ‘isolated’ *commune* group, the influence of population density was less clear-cut. However, a striking association with the weighted average migration distance was observed with a population density greater than >45  inh km^−2^ (SIRR=1.25 [0.97; 1.61] and SIRR=1.72 [1.23; 2.39] for a distance of 82–185 km and over 185 km, respectively). The results observed in the ‘non-isolated’ group were quite similar to those observed overall, but of slightly smaller magnitude and at only borderline statistical significance.

When the analyses were restricted to common B-cell ALL, the results were quite similar but the role of the population density in ‘isolated’ *communes* became less clear (not shown). Although there were slight inconsistencies, the analyses for the two subperiods, 1990–1996 and 1997–2003, favoured a positive association between CL incidence and population movements. Using linear interpolation between the two censuses to estimate the populations on the *commune* scale between 1990 and 1999 and over 1999 did not change the results. Similarly, the results were unchanged when merged or split (about 200) *communes* were excluded.

## DISCUSSION

Nationally, CL incidence was associated with the level of migrants in ‘isolated’ *communes*, more markedly above a given threshold of population density. Although of smaller magnitude, similar associations were seen in low-density ‘non-isolated’ *communes*. The associations were also more pronounced at ages 0–4 years, but were not specific to common B-cell ALL. Findings in the two subperiods were mostly similar, more marked in 1997–2003. Sensitivity analyses showed stability of the results with respect to the population estimates on the *commune* scale and their changes over time.

The present study examined the data for 14 years of national CL registration, judged as 99.2% complete (Clavel *et al*, 2004), so missing data were too small (0.2%) to have affected the results. It also benefited from INSEE's provision of details by urban status, and between-census population movements. The 10-year interval between censuses may have allowed any rapid and temporary changes in *communes* (e.g., wartime evacuation) to go undetected and introduce misclassifications, but there is no obvious reason why these would be differential and related to migration distance. An increase in the total population may be more important under the population mixing hypothesis than a sustained high proportion of migrants. The measures of population movements considered in the present study, mostly based on changes in address between 1990 and 1999, provided no indication of whether a high level of migrants was new. Any of these areas with a substantial proportion of migrants at some time before 1990 would have been considered with areas where the arrival of migrants was new, although they may not be strongly expected to show an excess following the arrival of other migrants between 1990 and 1999.

With regard to the infectious hypothesis, population movements may have a greater impact when they occur around the time of birth. Using place of residence at diagnosis may therefore be less accurate and might partly explain the weaker associations than those of Rudant *et al* (2006).

Many investigations have been conducted of CL incidence in rural isolated areas that had been subject to extreme and unusual population influxes (Kinlen *et al*, 1990, 1993, 2002; Kinlen and Hudson, 1991; Kinlen and John, 1994; Kinlen and Petridou, 1995; Kinlen, 1988, 2006; Dickinson and Parker, 1999; Koushik *et al*, 2001; Boutou *et al*, 2002; Wartenberg *et al*, 2004). Those studies, based on specific rural population increases, were mainly consistent in finding an increased risk of CL with an increasing proportion of incomers.

Unlike those studies that focused on new population increases, the present study considered the migrants who moved from a *commune* to another between the last two censuses with no account of whether the high levels of migration were new. Besides, the population variations between the census years were so skewed on the *commune* scale that this measure could not be considered in the present study.

Several other studies have shown that areas with high proportions of migrants or marked changes in population were at a significantly higher risk of CL than other areas (Langford, 1991; Stiller and Boyle, 1996; Alexander *et al*, 1997; Dickinson *et al*, 2002; Labar *et al*, 2004; Nyari *et al*, 2006; Rudant *et al*, 2006). In the UK, Dickinson *et al* found an increased risk of disease only in urban census wards. Two large studies did not demonstrate any significant association with the overall proportion of people who changed address in the year before a census (not specifically in rural areas), but found instead a negative association with an index of mixing diversity (Parslow *et al*, 2002; Law *et al*, 2003).

In the present study, a positive nonsignificant association was found only for the subgroup of isolated *communes* with a low population density. The highly skewed distribution of the proportions of migrants, with an inter-quartile distance of about 10%, could have hampered detection of a potential association. Moreover, considering the overall proportion of individuals who moved from one *commune* to another, regardless of distance, may be too imprecise a measure.

Only three studies have investigated migration distance in relation to CL, either directly (Stiller and Boyle, 1996) or by considering the proportions of migrants from distant areas (other district, other region, outside the country) (Dickinson *et al*, 2002; Rudant *et al*, 2006); all found a positive association with migration distance.

We considered migration distance using three measures that were significantly correlated. The further the migrants came, the higher was CL incidence, particularly at ages 0–4 years in ‘isolated’ areas. These results were in line with the literature and provide some support for the infectious hypothesis of Kinlen. This involvement of migration distance may reflect the fact that the further the migrants had travelled, it was less likely that there was much previous contact with the local population and therefore of having a similar immune status with respect to the hypothetical viral agent.

The associations were found in both isolation status groups but only in *communes* with a population density below 394 inh km^−2^ for the ‘non-isolated’ *commune* group. This may stem from possible inaccuracy in the definition of the isolation status. C*ommunes* that were located in the vicinity of an urban area, and thus dependent on it for employment, were considered ‘non-isolated’, regardless of their population size or population density. It was considered, perhaps erroneously, that the people who lived in those *communes* were likely to share a common immunity with the people who lived in the urban pole. If the association between CL incidence and migration distance was mainly in rural or isolated locations, as implied by Kinlen, the results found in the ‘non-isolated’ *communes* could reflect misclassification with respect to the real isolation status of the *communes*.

The involvement of population density may also indicate that the evaluation of the isolation status of French *communes*, which was intended to identify communities with low background immunisation, may not have been sufficiently pertinent to the infectious hypothesis.

However, a striking increase in CL incidence was found in relation to the weighted average migration distance in the ‘isolated’ group, but only in *communes* with a density greater than 45 inh km^−2^. This result, in line with the results of a French birth cohort study (Rudant *et al*, 2006), seems to favour a role for population density. There may be a threshold below which the suspected infection cannot spread because of the limited number of person-to-person contacts.

As in a previous study (Stiller and Boyle, 1996), no association with the proportion of commuters in the *communes* was found, although the definitions of commuting were slightly different. An investigation of increases of commuting in relation to CL found an 80% excess in locations with the greatest increases (Kinlen *et al*, 1991). With respect to the infectious hypothesis, an increase in commuting patterns over time may be more important than a sustained high level.

In contrast to Kinlen's work, based on specific rural influxes that increased total population and therefore its density, our study was based on migration measures (irrespective of population increases) over a 10-year period on a national scale, and took account of both isolation status and population density of the *communes*. The association of population movements with CL incidence in young children in ‘isolated’ areas at the time of diagnosis is consistent with the possible involvement of viral agents in this disease.

## Figures and Tables

**Table 1:** Distribution of the migration distance and distribution of the proxy measures of population mixing, for all *communes* and by ‘isolation’ status (see text) (max: maximum; min: minimum; Q1: first quartile; Q3: third quartile)

	**Mean**	**Min**	**Q1**	**Median**	**Q3**	**Max**
*Migration distance*						
All communes	196.6	0.0	25.3	95.4	328.4	1897.9
‘Isolated’ *communes*	160.7	0.0	17.8	58.2	247.0	1370.1
‘Non-isolated’ *communes*	212.2	0.0	30.2	117.5	357.0	1897.9
						
*Proxy measures of population mixing*						
Proportion of migrants (%)						
All communes	31.9	0.0	26.3	31.9	37.5	100.0
‘Isolated’ *communes*	30.5	0.0	24.7	30.2	36.0	100.0
‘Non-isolated’ *communes*	34.3	0.0	29.1	34.1	39.2	79.4
						
Proportion of migrants from another département (%)						
All communes	12.7	0.0	7.3	11.4	16.6	64.1
‘Isolated’ *communes*	13.2	0.0	7.9	12.1	17.2	64.1
‘Non-isolated’ *communes*	11.8	0.0	6.6	10.3	15.4	59.4
						
Proportion of migrants from another région (%)						
All communes	9.1	0.0	4.8	7.8	11.9	63.4
‘Isolated’ *communes*	9.8	0.0	5.3	8.7	13.0	63.4
‘Non-isolated’ *communes*	8.0	0.0	4.2	6.8	10.1	58.6
						
Proportion of migrants from distant^a^ communes (%)						
All communes	7.6	0.0	3.8	6.5	10.3	62.5
‘Isolated’ *communes*	8.3	0.0	4.1	7.3	11.3	62.5
‘Non-isolated’ *communes*	6.5	0.0	3.5	5.6	8.7	58.6
						
Weighted average migration distance (km)						
All communes	98.6	1.6	54.4	81.1	126.7	1269.7
‘Isolated’ *communes*	109.7	1.6	60.1	93.0	144.1	898.8
‘Non-isolated’ *communes*	80.9	2.4	48.7	68.1	97.8	1269.7
						
Proportion of commuters (%)						
All communes	39.9	0.0	30.5	37.7	45.1	2664.0
‘Isolated’ *communes*	34.8	0.0	27.1	33.1	39.5	1393.8
‘Non-isolated’ *communes*	47.9	12.9	38.8	44.0	50.7	2663.6

a ⩾100 km for all *communes*; ⩾60 km for ‘isolated’ *communes* and ⩾120 km for ‘non-isolated’ *communes*.

**Table 2:** Incidence of CL at ages 0–14 years over the period 1990–2003 and population movements in the French *communes* between 1990 and 1999, for all destination *communes* and by ‘isolation’ status (see text)

	**All communes**			**‘Isolated’ *communes***			**‘Non-isolated’ *communes***		
	**E**	**SIRR**	**95% CI**	**E**	**SIRR**	**95% CI**	**E**	**SIRR**	**95% CI**
*All migrants*									
⩽30%	2584.9	1	Ref.	491.6	1	Ref.	2092.2	1	Ref.
⩽38%	2483.9	1.05	[0.99–1.11]	408.3	1.06	[0.93–1.21]	2075.6	1.04	[0.98–1.11]
>38%	1205.4	0.98	[0.92–1.05]	167.8	1.05	[0.88–1.25]	1037.6	0.97	[0.9–1.04]
									
*Migrants from another département*									
⩽12%	2424.8	1	Ref.	511.2	1	Ref.	1912.6	1	Ref.
⩽21%	2554.4	1.01	[0.96–1.07]	432.2	1.03	[0.9–1.17]	2122.2	1.00	[0.94–1.07]
>21%	1294.9	0.99	[0.93–1.06]	124.3	1.06	[0.87–1.29]	1170.5	0.98	[0.91–1.06]
									
*Migrants from another région*									
⩽7%	2407.8	1	Ref.	367.8	1	Ref.	2039.0	1	Ref.
⩽13%	2731.4	1.01	[0.96–1.07]	453.7	1.09	[0.95–1.26]	2277.8	1	[0.94–1.06]
>13%	1134.8	1.08	[1.01–1.16]	246.2	1.18	[1.01–1.39]	888.6	1.06	[0.98–1.15]
									
*Migrants from distant*^a^ *communes*									
⩽L1^b^	2765.1	1	Ref.	388.7	1	Ref.	2079.8	1	Ref.
⩽L2^c^	2237.8	1.06	[1–1.12]	447.4	1.02	[0.89–1.18]	2023.3	1.02	[0.96–1.09]
>L2^c^	1271.2	1.08	[1.02–1.16]	231.6	1.14	[0.97–1.33]	1102.2	1.08	[1–1.16]
									
*Weighted average migration distance*^d^ *(km)*									
⩽82	2507.7	1	Ref.	421.3	1	Ref.	2085.4	1	Ref.
⩽185	2775.6	1.05	[1–1.11]	513.3	1.17	[1.02–1.34]	2262.2	1.03	[0.97–1.09]
>185	990.8	1.10	[1.02–1.18]	133.1	1.24	[1.02–1.5]	857.7	1.07	[0.99–1.16]
									
*Commuters*									
⩽L3^e^	2424.1	1	Ref.	405.7	1	Ref.	2124.5	1	Ref.
⩽L4^f^	2559.9	1.03	[0.97–1.09]	442.1	1.13	[0.98–1.29]	1949.1	0.96	[0.9–1.02]
>L4^f^	1260.0	1.01	[0.94–1.08]	219.8	1.10	[0.93–1.3]	1131.7	0.98	[0.91–1.05]

E=expected number of cases; SIRR=SIR (standardised incidence ratio) ratio.

SIRR estimated by Poisson's regression.

a ⩾100 km for all *communes;* ⩾60 km for ‘isolated’ *communes* and ⩾120 km for ‘non-isolated’ *communes.*

b 0.07 for all *communes*; 0.08 for ‘isolated’ *communes*; 0.06 for ‘non-isolated’ *communes.*

c 0.12 for all *communes*; 0.14 for ‘isolated’ *communes*; 0.11 for ‘non-isolated’ *communes*.

d Adjusted for the proportion of migrants.

e 0.42 for all *communes*; 0.33 for ‘isolated’ *communes*; 0.45 for ‘non-isolated’ *communes*.

f 0.59 for all *communes*; 0.44 for ‘isolated’ *communes*; 0.60 for ‘non-isolated’ *communes*.

**Table 3:** Incidence of CL at ages 0–4 years over the period 1990–2003 and population movements in the French *communes* between 1990 and 1999, for all destination *communes* and by ‘isolation’ status (see text)

	**All communes**			**‘Isolated’ communes**			**‘Non-isolated’ communes**		
	**E**	**SIRR**	**95% CI**	**E**	**SIRR**	**95% CI**	**E**	**SIRR**	**95% CI**
*All migrants*									
⩽30%	1293.5	1	Ref.	233.5	1	Ref.	1059.5	1	Ref.
⩽38%	1237.4	1.05	[0.97–1.13]	199.5	1.06	[0.88–1.28]	1037.9	1.04	[0.96–1.13]
>38%	599.3	1.02	[0.92–1.12]	83.1	1.15	[0.9–1.46]	516.2	0.99	[0.89–1.11]
									
*Migrants from another département*									
⩽12%	1171.3	1	Ref.	245.4	1	Ref.	925.4	1	Ref.
⩽21%	1277.6	0.98	[0.91–1.06]	210.3	0.99	[0.83–1.19]	1067.3	0.98	[0.9–1.07]
>21%	681.3	0.97	[0.88–1.06]	60.5	1.09	[0.84–1.43]	620.8	0.95	[0.86–1.06]
									
*Migrants from another région*									
⩽7%	1177.9	1	Ref.	176.2	1	Ref.	1001.2	1	Ref.
⩽13%	1380.8	1.00	[0.93–1.09]	220.3	1.07	[0.88–1.31]	1160.5	0.99	[0.91–1.08]
>13%	571.5	1.09	[0.99–1.21]	119.7	1.20	[0.95–1.5]	451.8	1.07	[0.96–1.19]
									
*Migrants from distant*^a^ *communes*									
⩽L1^b^	1353.5	1	Ref.	186.4	1	Ref.	1021.3	1	Ref.
⩽L2^c^	1132.0	1.05	[0.97–1.13]	217.7	0.95	[0.78–1.15]	1029.9	1.02	[0.94–1.12]
>L2^c^	644.7	1.11	[1.02–1.22]	112.2	1.25	[1.01–1.56]	562.3	1.09	[0.98–1.21]
									
*Weighted average migration distance* d *(km)*									
⩽82	1223.1	1	Ref.	203.8	1	Ref.	1018.8	1	Ref.
⩽185	1397.1	1.06	[0.98–1.14]	248.5	1.19	[0.99–1.44]	1148.6	1.03	[0.95–1.12]
>185	510.0	1.14	[1.03–1.26]	63.9	1.41	[1.09–1.83]	446.1	1.09	[0.98–1.22]
									
*Commuters*									
⩽L3^e^	1208.9	1	Ref.	194.3	1	Ref.	1053.9	1	Ref.
⩽L4^f^	1270.5	0.98	[0.91–1.06]	214.4	1.12	[0.93–1.36]	973.5	0.9	[0.82–0.98]
>L4^f^	650.8	0.96	[0.87–1.05]	107.5	1.17	[0.93–1.48]	586.1	0.93	[0.84–1.03]

E=expected number of cases; SIRR=SIR (standardised incidence ratio) ratio.

SIRR estimated by Poisson regression.

a ⩾100 km for all *communes;* ⩾60 km for ‘isolated’ *communes* and ⩾120 km for ‘non-isolated’ *communes*.

b 0.07 for all *communes*; 0.08 for ‘isolated’ *communes*; 0.06 for ‘non-isolated’ *communes*.

c 0.12 for all *communes*; 0.14 for ‘isolated’ *communes*; 0.11 for ‘non-isolated’ *communes*.

d adjusted for the proportion of migrants.

e 0.42 for all *communes*; 0.33 for ‘isolated’ *communes*; 0,45 for ‘non-isolated’ *communes*.

f 0.59 for all *communes*; 0.44 for ‘isolated’ *communes*; 0,60 for ‘non-isolated’ *communes*.

**Table 4:** Incidence of CL at ages years 0–4 over the period 1990–2003 and population movements between 1990 and 1999 with stratification by the 1990 population density of the *communes*

	**Low density**			**Medium density**			**High density**		
	**E**	**SIRR**	**95% CI**	**E**	**SIRR**	**95% CI**	**E**	**SIRR**	**95% CI**
*All communes*	⩽180 inh km^−2^			180–1729 inh km^−2^			>1729 inh km^−2^		
Migrants from another région									
⩽7%	382.3	1.00	Ref.	367.4	1.00	Ref.	427.7	1.00	Ref.
⩽13%	421.3	1.10	[0.95–1.27]	422.0	0.96	[0.84–1.10]	537.5	0.96	[0.85–1.10]
>13%	190.9	1.27	[1.07–1.51]	215.1	1.01	[0.86–1.19]	165.5	1.01	[0.84–1.21]
									
Migrants from a distant commune (>100 km)									
⩽7%	480.7	1.00	Ref.	433.0	1.00	Ref.	439.3	1.00	Ref.
⩽12%	340.7	1.06	[0.92–1.22]	345.3	1.09	[0.95–1.26]	446.0	0.99	[0.87–1.14]
>12%	173.1	1.22	[1.03–1.45]	226.2	1.04	[0.89–1.22]	245.4	1.09	[0.94–1.28]
									
Weighted average migration distance (km)									
⩽82	454.9	1.00	Ref.	404.4	1.00	Ref.	363.4	1.00	Ref.
⩽185	438.4	1.12	[0.98–1.28]	457.9	1.06	[0.93–1.21]	500.8	1.00	[0.87–1.14]
>185	101.2	1.28	[1.04–1.58]	142.3	1.09	[0.91–1.31]	266.5	1.09	[0.91–1.25]
									
*‘Non-isolated’ communes*	≤394 inh km^−2^			394–2607 inh km^−2^			>2607 inh km^−2^		
Migrants from another région									
⩽7%	323.0	1.00	Ref.	335.1	1.00	Ref.	343.0	1.00	Ref.
⩽13%	345.6	1.02	[0.87–1.19]	348.8	0.97	[0.84–1.12]	466.1	1.00	[0.86–1.15]
>13%	149.2	1.19	[0.99–1.44]	166.3	0.96	[0.80–1.15]	136.4	1.08	[0.88–1.31]
									
Migrants from a distant commune (>120 km)									
⩽7%	353.3	1.00	Ref.	355.4	1.00	Ref.	312.6	1.00	Ref.
⩽12%	302.1	1.04	[0.89–1.21]	306.7	1.03	[0.88–1.19]	421.1	1.02	[0.88–1.19]
>12%	162.4	1.19	[0.99–1.43]	188.1	0.99	[0.83–1.18]	211.7	1.11	[0.93–1.32]
									
Weighted average migration distance (km)									
⩽82	388.6	1.00	Ref.	332.8	1.00	Ref.	297.5	1.00	Ref.
⩽185	338.2	1.06	[0.92–1.23]	408.9	1.02	[0.88–1.18]	401.5	1.02	[0.87–1.19]
>185	91.1	1.21	[0.98–1.51]	108.6	0.98	[0.79–1.21]	246.4	1.10	[0.93–1.3]
									
*‘Isolated’ communes*	⩽45 inh km^−2^			>45 inh km^−2^					
Migrants from another region									
⩽7%	83.8	1.00	Ref.	92.4	1.00	Ref.			
⩽13%	110.5	1.04	[0.76–1.41]	109.8	1.11	[0.86–1.44]			
>13%	66.4	1.27	[0.91–1.77]	53.3	1.17	[0.86–1.60]			
									
Migrants from a distant commune (>60 km)									
⩽8%	88.6	1.00	Ref.	97.8	1.00	Ref.			
⩽14%	109.2	0.95	[0.7–1.29]	108.5	0.95	[0.73–1.24]			
>14%	62.9	1.21	[0.87–1.68]	49.3	1.35	[1.00–1.81]			
									
Weighted average migration distance (km)									
⩽82	99.7	1.00	Ref.	104.1	1.00	Ref.			
⩽185	129.2	1.14	[0.86–1.50]	119.3	1.25	[0.97–1.61]			
>185	31.8	1.09	[0.71–1.66]	32.1	1.72	[1.23–2.39]			
